# Health literacy in patients with gout: A latent profile analysis

**DOI:** 10.1371/journal.pone.0300983

**Published:** 2024-05-09

**Authors:** Shuo Cai, Danqing Hu, Derong Wang, Jianchun Zhao, Haowei Du, Aimin Wang, Yuting Song

**Affiliations:** School of Nursing, Qingdao University, Qingdao, Shandong Province, People’s Republic of China; Xiamen University - Malaysia Campus: Xiamen University - Malaysia, MALAYSIA

## Abstract

**Objectives:**

Understanding the health literacy status of patients with gout diagnosis is essential for improving the health of this population. Our study aimed to investigate the latent profiles of health literacy in patients with gout and to analyze differences in characteristics across potential profiles.

**Methods:**

This was a cross-sectional study. Eligible participants attended the Shandong Gout Medical Center, from March 2023 to May 2023 and self-reported gout diagnosis. We used the Health Literacy Scale for Patients with Gout designed and validated by our team. The scale had good reliability and validity among patients with gout. 243 patients completed the Demographic Information Questionnaire and the Health Literacy Scale for Patients with Gout. We used latent profile analysis to identify the latent profiles of gout patients’ health literacy. We used Chi-square tests with Bonferroni correction to analyze differences in demographics and illness characteristics across identified profiles.

**Results:**

Three profiles of patients with gout emerged (prevalence): the *low literacy-low critical* group (21.81%), the *moderate literacy* group (42.79%), and the *high literacy-stable* group (35.39%). The three groups differed in age, education level, monthly income, disease duration, and place of residence (*P*<0.01).

**Conclusions:**

The health literacy of patients with gout was heterogeneous. Healthcare professionals should adopt targeted interventions based on the characteristics of each latent health literacy profile to improve the health literacy level of patients with gout.

## Introduction

Gout is a metabolic disease caused by a disorder of purine metabolism or impaired excretion of uric acid, resulting in the deposition of urate crystals [1]. The global prevalence of gout ranges from <1% to 6.8% across countries, and the prevalence in China is about 1.1% [2]. Globally, the incidence of gout is on the rise and has become one of the most common and frequent diseases that jeopardize the health of the population [2]. Patients with gout usually suffer from pain, joint deformity, impaired mobility, kidney damage, anxiety, depression, and other problems, which seriously jeopardize the physical and mental health and quality of life of patients with gout [3, 4]. As a chronic disease, gout is prone to recurrent flares (when symptoms get worse), and patients with gout need to take action to manage the disease over the long term to improve their health outcomes [5]. However, the current health behaviors and health outcomes of patients with gout need to be improved. For example, healthcare utilization and medication adherence among this patient population are insufficient [6, 7]. The health behaviors and health outcomes of patients with chronic diseases are strongly influenced by patients’ health literacy, referring to people’s cognitive and social skills and ability to access, understand, and apply health information to improve their health [8]. Studies have shown that adequate health literacy is associated with higher health-related quality of life [9, 10]. In contrast, limited health literacy can lead to reduced access and utilization of healthcare services, decreased medication adherence, and poor self-management [11–13]. The health literacy of patients with gout needs to be improved, and there is a need to focus on the health literacy of patients with gout [11].

Nutbeam’s health literacy theory covers all aspects of health literacy and is widely used in the health literacy field [14, 15]. Nutbeam [16] proposed three dimensions of health literacy: functional, interactive, and critical. Functional health literacy occurs when patients have specific literacy skills and knowledge of chronic diseases; interactive health literacy refers to patients’ ability to actively obtain, communicate, and use health information through various means; and critical health literacy refers to patients’ ability to use critical thinking to critically analyze chronic disease-related information according to one’s actual situation [16]. However, most of the current scales used to measure health literacy in patients with gout do not cover functional, interactive, and critical aspects and cannot reflect the characteristics of patients with gout comprehensively and in detail [11, 17]. Moreover, disease-specific health literacy assessment instruments are considered more beneficial for clinical care [18], whereas most of the current scales used to measure health literacy in patients with gout are generic scales [17, 19]. The use of a disease-specific health literacy scale for patients with gout that covers functional health literacy, interactive health literacy, and critical health literacy may help to better understand the health literacy of patients with gout.

Most studies assessing the health literacy of patients with chronic diseases have used a variable-centered approach. The studies have widely investigated the factors that influence health literacy of patients with chronic diseases. Researchers have reported that demographic characteristics (e.g., age, education level, income) and disease factors (e.g., number of diseases, duration of illness) are associated with health literacy among patients with chronic diseases [20–24]. However, previous research has shown that health literacy is complex and perhaps heterogeneous [25], and the variable-centered approach does not take into account individual heterogeneity, suggesting a person-centered approach [26] is warranted. Latent profile analysis is a person-centered approach that groups individuals homogeneously based on differences in their scores on each variable (dimensions of health literacy), which is conducive to studying the characteristics of different types of populations [26, 27]. This approach categorizes individuals into different groups to help target interventions to different subgroups, which can lead to better intervention outcomes, especially for disadvantaged patients [28, 29]. Therefore, in this study, we surveyed patients with gout using our team-designed Health Literacy Scale, which covers the three dimensions of Nutbeam’s Health Literacy Theory and is a disease-specific scale. We aimed to draw on the latent profile analysis to investigate the latent profiles of health literacy in patients with gout and analyze differences in characteristics across latent profiles to provide a basis for further development of targeted interventions.

## Materials and methods

### Participants

#### Sampling and recruitment

We recruited patients from the Shandong Gout Medical Center, using a convenience sampling method. The Shandong Gout Medical Center is located in Qingdao, Shandong Province, a coastal city in northeastern China with an ample number of patients with gout. Our research team has established long-term good research cooperation with Shandong Gout Medical Center.

#### Inclusion criteria

Eligible participants: (a) met the American College of Rheumatology diagnostic criteria for gout [30] (b) were aged 18years or older, and (c) were able to read and understand the questionnaire.

#### Exclusion criteria

We excluded people who (a) suffered from other serious acute or chronic diseases, such as malignant tumors, severe hearing impairments, or severe mental illnesses, or(b) dropped out or answered the questionnaire incompletely.

#### Sample size

We adopted the common-used approach that the sample size should be 5–10 times the number of independent variables [31]. The number of independent variables in this study was 11, and taking into account the 10%-20% null rate, the actual sample size of this study was 243 cases.

### Operating definition

Synthesizing the World Health Organization’s definition of health literacy [8] and Nutbeam’s health literacy theory [16], the health literacy of patients with gout is defined as the gout patient’s mastery of gout-related knowledge and the ability to access, understand, communicate, apply and critically analyze gout-related information.

### General characteristics

General characteristics of patients included patients’ demographic and illness-related characteristics. The following categorical variables were reported: age, gender, marital status, education level, monthly income, place of residence, BMI, duration of gout, tophi, a family history of gout, and other chronic disease conditions.

### Health literacy for patients with gout

We assessed the health literacy of patients with gout using a self-developed Health Literacy Scale. Our research team developed a health literacy scale that meets the characteristics of patients with gout. Using the Nutbeam health literacy model [14] as a theoretical guide, our research team developed the Health Literacy Scale for Patients with Gout after referencing other published studies on health literacy in patients with gout [31–35], conducting semi-structured interviews with 13 patients with gout, two rounds of correspondence with 18 Delphi experts, and testing the reliability and validity of the scale with 481 participants (S2 File).

The scale consists of 31 entries in 4 dimensions. They are basic knowledge functional health literacy (5 entries), self-care knowledge functional health literacy (15 entries), interactive health literacy (7 entries), and critical health literacy (4 entries). Basic knowledge functional health literacy refers to basic medical knowledge related to gout, including the knowledge of the pathogenesis, complications, staging, heredity, and uric acid attainment value of gout. Self-care knowledge functional health literacy refers to gout-related self-care knowledge, including knowledge of diet, medication, exercise, and pain management. Interactive health literacy involves acquiring, understanding, and applying gout-related health information. Critical health literacy refers to judgments about gout-related health information, including judgments about the correctness, authority, and applicability of health-related information. In the basic knowledge functional health literacy and self-care knowledge functional health literacy sections, each item is scored on a 5-point Likert scale ranging from “1 = I don’t know at all” to “5 = I know fully.” In the interactive health literacy and critical health literacy sections, each item is scored on a 5-point Likert scale ranging from “1 = not at all” to “5 = always.” The total score on the scale ranged from 31 to 155, with higher scores indicating higher levels of health literacy. The Cronbach’s alpha coefficient of the scale was 0.972, the split-half reliability was 0.925, and the re-test reliability after two weeks was 0.934. The scale-level content validity index was 0.903, and the item-level content validity index was 0.833~1.000.

### Data collection

This study was approved by the Ethics Committee of Qingdao University Medical Department (QDU-HEC-2022211). The data collection period was from March 6, 2023, to May 30, 2023. The investigators contacted the medical staff at the Gout Medical Center beforehand. The investigators then recruited patients to participate in this survey with the assistance of the medical staff at the Gout Medical Center. The investigators introduced the purpose and content of this study to the patients. After obtaining written informed consent from the patients, the investigators distributed the questionnaires and told them what to look for when completing the questionnaires. Demographic information was patient-reported, and disease-related information was completed by the researcher from the patient’s medical records. For patients who had difficulty answering the questionnaire independently, the investigator read out the questionnaire items to the patients and filled in the questionnaire according to the patient’s answers. After completing the questionnaire, we gave the patients a thank-you gift. A total of 254 questionnaires were distributed in this study, and 243 valid questionnaires were recovered, with an effective recovery rate of 95.67%.

### Data analysis

Latent profile modeling was performed using the Mplus 8.3 software. We used the four dimensions of health literacy of patients with gout as the exogenous variable. The initial model started with one profile and gradually increased the number of profiles to determine the optimal fitting model. Fit indicators included the Akaike information criterion (AIC), Bayesian information criterion (BIC), sample size‐adjusted Bayesian information criterion (aBIC), entropy, Lo-Mendell-Rubin adjusted likelihood ratio test (LMR), and Bootstrap Likelihood Ratio Test (BLRT) [36, 37]. The smaller the value of AIC, BIC, and aBIC, the better the model fit. The entropy range is 0~1, and the entropy value greater than 0.76 represents high classification accuracy [38]. Lo-Mendell-Rubin adjusted likelihood ratio test (LMR) and bootstrap likelihood ratio test (BLRT) indicate the difference between different potential profile models. P<0.05 suggests that the current model fit is better than the previous model.

Data analysis was performed using the SPSS 25.0 software. We used mean and standard deviation to describe continuous data following normal distributions and frequency and percentage (%) to describe count data. Differences in characteristics across latent profiles were performed using Chi-squares tests with Bonferroni correction. Statistical significance in this study is indicated by P<0.05.

## Results

### Participant characteristics

The participants were predominantly under 60 years of age, male, married, and had completed high school or more education. Of the participants, 138 (56.79%) had a monthly income of less than RMB 6000 (which is approximately 827 US dollars), 172 (70.78%) lived in the urban area, and 120 (49.38%) had been diagnosed with gout for five or more years. Forty patients (16.87%) had a family history of gout, and 89 (36.62%) patients also lived with other chronic diseases (Table 1).

**Table 1:** Participant characteristics (n = 243).

Variables		n (%)
**Age (year)**	<60	186 (76.54)
	≥60	57 (23.46)
**Gender**	Male	223 (91.77)
	Female	20 (8.23)
**Marital status**	Single, divorced, or widowed	49 (20.16)
	Married	194 (79.84)
**Education level**	Junior high school or below	77 (31.69)
	High school or above	166 (68.31)
**Income/month (RMB)**	<6000	138 (56.79)
	≥6000	105 (43.21)
**Place of residence**	Rural	71(29.22)
	Urban	172 (70.78)
**BMI (kg/m** ^**2**^ **)**	18.50~23.90	56 (23.05)
	24.00~27.90	104 (42.80)
	≥28.00	83 (34.16)
**Duration of gout (year)**	<5	123 (50.62)
	≥5	120 (49.38)
**Tophi**	No	149 (61.32)
	Yes	94 (38.68)
**A family history of gout**	No	202 (83.13)
	Yes	41 (16.87)
**Other chronic diseases**	No	154 (63.37)
	Yes	89 (36.62)

Note: 6,000 RMB is approximately 827 US dollars.

### Classification of latent profiles of health literacy

The model fit indices for latent profile analysis of health literacy are shown in Table 2. Models with 1 to 5 potential profiles were constructed stepwise with the four dimensions of gout patients’ health literacy entries averaged as exogenous variables. As the number of profiles increased, the AIC, BIC, and aBIC values gradually decreased. However, the Entropy value for the 3-profile model was higher than the 4-profile model, and both the LMR value and BLRT value reached significant levels for the 3-profile model. Collectively, the 3-profile model emerged as the best model fitting the data, indicating three latent profiles of patients with gout by their health literacy.

**Table 2:** Model fit indices for latent profile analysis of health literacy (n = 243).

Number of profiles	AIC	BIC	aBIC	Entropy	LMR (*P* value)	BLRT (*P* value)	Latent Profile Proportion
1	2664.581	2692.525	2667.166	-	-	-	1
2	2351.380	2396.790	2355.582	0.816	**<0.001**	**<0.001**	0.531/0.469
3	2265.045	2327.920	2270.863	0.785	**0.001**	**<0.001**	0.218/0.428/0.354
4	2239.129	2319.470	2246.563	0.755	0.366	**<0.001**	0.256/0.272/0.325/0.148
5	2222.901	2320.707	2231.951	0.792	0.773	<**0.001**	0.103/0.230/0.193/0.300/0.173

**Fig 1:**
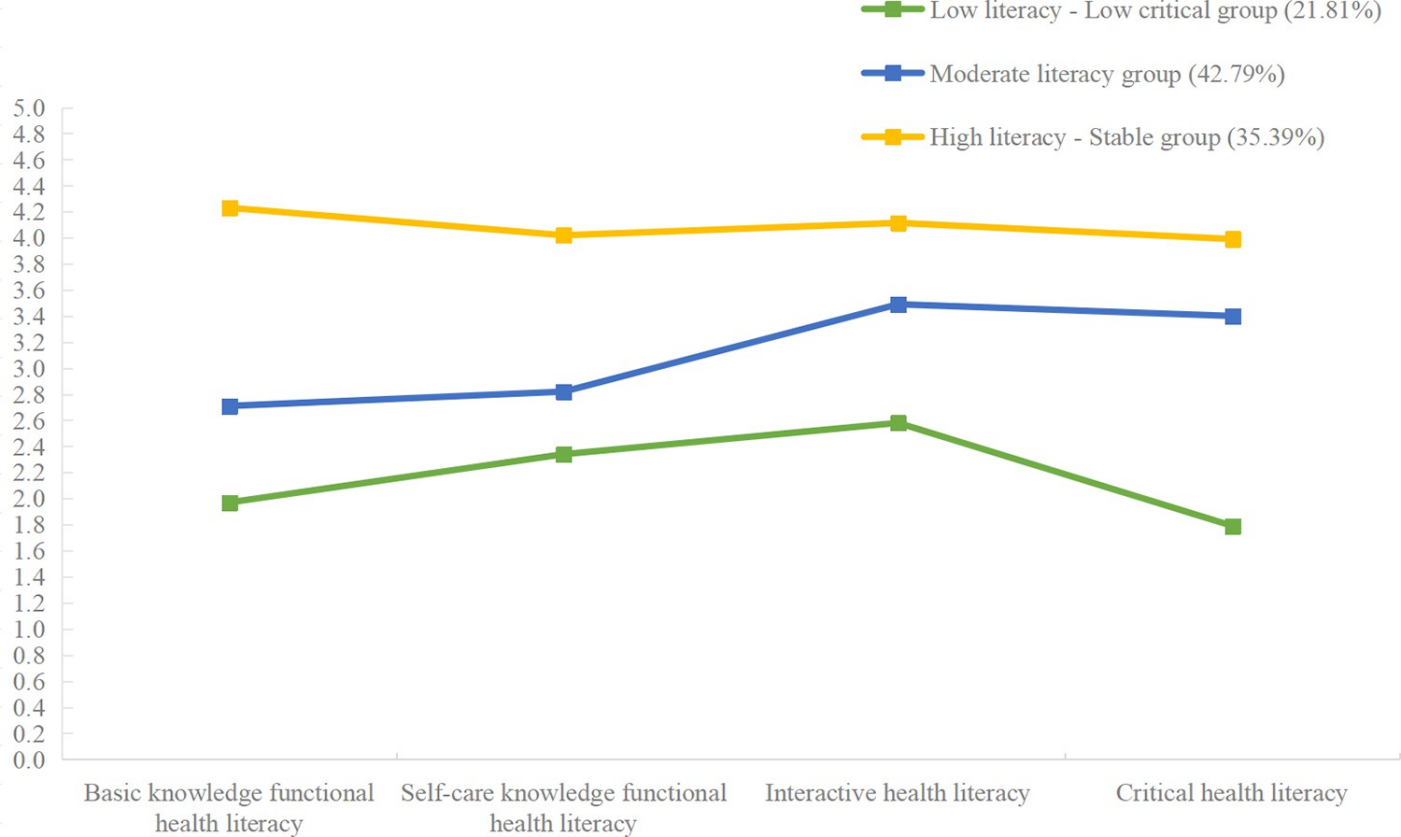
Mean scores for each dimension of the three-profile model of health literacy (n = 243).

The results of the latent profile analysis of health literacy among patients with gout are shown in Fig 1 and Table 3. The first profile consisted of 21.81% (53). The profile’s mean scores on all dimensions were lower than the other groups, and the critical health literacy dimension mean scores were lower than the other dimensions. Therefore, it was named the *low literacy-low critical* group. The second profile consisted of 42.79% (104). The profile’s mean scores on all dimensions were in the middle level compared to other groups. Therefore, it was named the *moderate literacy group*. The third profile consisted of 35.39% (86). The profile’s mean scores on all dimensions were higher than the other groups, and the difference in mean scores between the four dimensions was slight and less volatile. Therefore, it was named the *high literacy-stable* group.

**Table 3:** Mean scores and standard deviation for each dimension of the three-profile model of health literacy (n = 243).

	Profile 1	Profile 2	Profile 3
	Low literacy-Low critical group (n = 53)	Moderate literacy group (n = 104)	High literacy-Stable group (n = 86)
	M (SD)	M (SD)	M (SD)
**Basic knowledge functional health literacy**	1.97 (0.67)	2.71 (0.65)	4.25 (0.59)
**Self-care knowledge functional health literacy**	2.34 (0.62)	2.82 (0.59)	4.03 (0.60)
**Interactive health literacy**	2.58 (0.43)	3.49 (0.47)	4.10 (0.54)
**Critical health literacy**	1.79 (0.55)	3.40 (0.76)	3.99 (0.74)

Note: M = Mean; SD = Standard Deviation.

### Differences in demographics and disease characteristics among different health literacy profiles

The results showed statistically significant differences between the three groups regarding age, education level, monthly income, place of residence, and duration of illness (P<0.01). The *low literacy-low critical* (39.62%) group had a higher percentage of patients aged greater than or equal to 60 years than the *moderate literacy* (20.19%) and *high literacy-stable* (17.44%) groups. The *low literacy-low critical* (64.15%) group had a higher percentage of patients with junior high school or lower education levels than the *moderate literacy* (26.92%) and *high literacy-stable* (17.44%) groups. The *low literacy-low critical* (83.02%) and *moderate literacy* (66.35%) groups had a higher percentage of patients with monthly incomes of less than 6,000 than the *high literacy-stable* (29.06%) group. The *low literacy-low critical* (45.28%) and *moderate literacy* (41.35%) groups had a higher percentage of rural patients than the *high literacy-stable* (4.65%) group. The *low literacy-low critical* (73.58%) and *moderate literacy* (56.73%) groups had a higher proportion of patients with less than five years of disease duration than the *high literacy-stable* (29.06%) group (Table 4).

**Table 4:** Comparison of demographics and disease characteristics between different health literacy profiles (n = 243).

Variables		Profile 1	Profile 2 Moderate literacy group (n = 104)	Profile 3	χ^2^	*P* value	Multiple Comparison with *p<0*.*017*
		Low literacy-Low critical group (n = 53)		High literacy-Stable group (n = 86)			
		N(%)	N(%)	N(%)			
**Age (year)**	<60	32 (60.38)	83 (79.81)	71 (82.56)	10.065	**0.007**	PF1vs.PF2,PF3
	≥60	21 (39.62)	21 (20.19)	15 (17.44)			
**Gender**	Male	45 (84.91)	95 (91.35)	83 (96.51)	5.891	0.053	none
	Female	8 (15.09)	9 (8.65)	3 (3.49)			
**Marital status**	Single, divorced, or widowed	7 (13.21)	28 (26.92)	14 (16.27)	5.351	0.069	none
	Married	46 (86.79)	76(73.08)	72 (83.73)			
**Education level**	Junior high school or below	34 (64.15)	28 (26.92)	15 (17.44)	34.957	**<0.001**	PF1vs.PF2,PF3
	High school or above	19 (35.85)	76 (73.08)	71 (82.56)			
**Income/month (RMB)**	<6000	44 (83.02)	69 (66.35)	25 (29.06)	45.659	**<0.001**	PF3vs.PF1,PF2
	≥6000	9 (17.98)	35 (33.65)	61 (70.94)			
**Place of residence**	Rural	24 (45.28)	43 (41.35)	4 (4.65)	39.108	**<0.001**	PF3vs.PF1,PF2
	Urban	29 (54.72)	61 (58.65)	82 (95.35)			
**BMI (kg/m** ^**2**^ **)**	18.50~23.90	15 (28.30)	16 (15.38)	25 (29.07)	6.145	0.189	none
	24.00~27.90	21 (39.62)	48 (46.15)	35 (40.70)			
	≥28.00	17 (32.08)	40 (38.46)	26 (30.23)			
**Duration of gout (year)**	<5	39 (73.58)	59 (56.73)	25 (29.06)	28.714	**<0.001**	PF3vs.PF1,PF2
	≥5	14 (26.42)	9 (43.27)	61 (70.94)			
**Tophi**	No	29 (54.72)	70 (67.31)	50 (58.14)	2.913	0.233	none
	Yes	24 (45.28)	34 (32.69)	36 (41.86)			
**A family history of gout**	No	45 (84.91)	90 (86.54)	67 (77.91)	2.653	0.265	none
	Yes	8 (15.09)	14 (13.46)	19 (22.09)			
**Other chronic diseases**	No	29 (54.71)	69 (66.35)	56 (65.12)	2.222	0.330	none
	Yes	24 (45.29)	35 (33.65)	30 (34.88)			

Note: 6,000 RMB is approximately 827 US dollars.

## Discussion

Our study showed significant individual differences in health literacy among patients with gout, which could be categorized into three groups: the *low literacy-low critical* group (21.81%), the *moderate literacy* group (42.79%), and the *high literacy-stable* group (35.39%). The *low literacy-low critical* group had a low level of health literacy and especially lacked the ability to critically analyze information related to chronic diseases. The *moderate literacy* group had a middle level of health literacy and a slight lack of basic knowledge of gout and self-care knowledge. The *high literacy-stable* group had a high level of health literacy and could know basic gout knowledge and self-care knowledge. This group can better acquire, communicate, and apply health information and can use critical thinking to critically analyze chronic disease-related information. Healthcare professionals should recognize the differences between the health literacy enhancement needs of patients with different profiles and develop and implement targeted interventions for different profiles to improve the health literacy level of gout patients.

The identified profiles of health literacy among patients with gout inform targeted strategies for improving health literacy for patients in each group. For patients in the *low literacy-low critical* group, healthcare professionals should focus on helping patients in this group improve their overall health literacy while assisting them in developing critical thinking. Healthcare professionals could train this group of patients in targeted sessions to inform them of tips for information judgment and guide them to make correct judgments. Patients in the *moderate literacy* group appeared to be the most likely to benefit from interventions that focus primarily on improving patients’ functional health literacy. Healthcare professionals should focus on their health education and explain their knowledge about the disease. Mobile e-health technology can be effective in improving the knowledge of patients with gout and may be a good choice of approach for health education [39]. Moreover, healthcare professionals could motivate patients with gout to learn about gout by reinforcing the importance of the disease through motivational interviews and peer-to-peer communication sessions for patients with gout. In addition, proactive self-management has been shown to be associated with better health outcomes [40]. For patients in the *high literacy-stable* group, healthcare professionals could encourage them to actively participate in developing their personal diagnostic and treatment programs, giving full play to their subjective initiative to manage their disease. For example, healthcare professionals could encourage this group of patients to participate in the development of their personal diet and exercise programs.

Of the three profiles, the *low literacy-low critical* group requires particular attention from healthcare professionals given their demographic and disease characteristics. The majority of patients in this profile were older than or equal to 60 years of age, had junior high school or less education, had a monthly income of less than RMB 6000 (which is approximately 827 US dollars), lived in rural areas, and had a disease duration of less than five years. This is consistent with the findings of previous studies, which have shown that age, education level, monthly income, place of residence, and disease duration are associated with health literacy [24, 41–43]. Several reasons may explain why patients in this group performed the worst in all aspects of health literacy. Older patients and those with lower levels of education can face difficulties with accessing, understanding, and critically judging health information [44]. Also, patients with lower monthly incomes and those in rural areas usually have a heavier financial burden, a relatively poor accessible healthcare environment, and a lack of awareness of the disease [45, 46]. In the early stages of the disease, the patients may not understand the disease, and as the duration of the disease increases, the patients gain more knowledge and experience of the disease [47]. Therefore, healthcare professionals should focus on patients who are older, less educated, have lower monthly incomes, live in rural areas, and have a shorter duration of illness. Easy-to-read health education materials facilitate health literacy [48]. For these patients, healthcare professionals should create easy-to-understand health education materials to help patients better understand the health information. At the same time, a lack of reliable sources of health information could lead to a lack of health literacy [49]. Healthcare professionals could inform patients of some reliable sources of health information, such as authoritative books and websites so that they can find appropriate health information from reliable sources and develop their critical thinking. In addition, policymakers should improve the health insurance system to reduce patients’ disease burden. Moreover, policymakers should emphasize the fair distribution of medical resources and increase the health promotion efforts of primary medical structures.

### Study strengths

To our knowledge, this is the first study on the latent profile analysis of health literacy in patients with gout. Latent profile analysis, unlike the variable-centered approach, allows for differential grouping of individuals and facilitates further targeting of interventions to different subgroups of patients, especially for disadvantaged patients [27, 28]. Our study identified heterogeneity in the health literacy of patients with gout, who can be categorized into three subgroups. Of them, the patients in the *low literacy-low critical* group who were older, less educated, had lower monthly incomes, lived mainly in rural areas, and had shorter disease duration urgently require targeted interventions to focus on improving critical health literacy for this subgroup of patients. Identifying subgroups facilitates the development of targeted interventions to improve health literacy in patients with gout. We recommended further research to develop and evaluate effective interventions to improve health literacy for the identified subgroups of patients with gout. In addition, disease-specific health literacy assessment instruments are considered more useful for clinical practice [18]. Our study used a health literacy assessment instrument that is specific to patients with gout rather than a generic scale, which contributes to a more comprehensive and targeted investigation of the health literacy of patients with gout. Moreover, we followed a rigorous process when developing the Health Literacy Scale for Patients with Gout, which had high reliability and validity. This ensured the credibility and reliability of our findings.

### Study limitations

The study has several limitations. First, this study was a cross-sectional study and could not confirm the causal relationship between variables. Longitudinal observational studies of health literacy among patients with gout are needed to understand the dynamics of health literacy among patients with gout and clarify the causal relationship between variables. Second, the factors influencing health literacy among patients with gout included in this study were limited, and we did not include factors related to patient’s psychological and social aspects that might influence health literacy [50, 51]. Future studies are warranted to explore these factors that influence the health literacy of patients with gout. Third, although we followed a rigorous process when developing the Health Literacy Scale for Patients with Gout that had high reliability and validity, comparisons with other studies need to be made with caution due to the different research instruments. In addition, our sample was recruited from a medical center in urban China, and results may not be generalizable to patients with different demographic and clinical characteristics from our sample.

## Conclusions

Health literacy in patients with gout is individually heterogeneous. In our sample, three subtypes existed: *low literacy-low critical* group, *moderate literacy group*, and *high literacy-stable* group. The three subtypes differed in age, education level, monthly income, place of residence, and duration of illness. Healthcare professionals should develop and adopt targeted interventions according to the characteristics of different categories of patients to improve their health literacy.

## Supporting information

S1 File The dataset used in the manuscript. (XLSX)

S2 File Health literacy scale for patients with gout (English version). (DOCX)

S3 File Health literacy scale for patients with gout (Chinese version). (DOCX)
