# Supplementary material for: Health literacy in patients with gout: A latent profile analysis
Source: PLoS One. 2024 May 9;19(5):e0300983. doi: 10.1371/journal.pone.0300983 (PMC11081339; doi:10.1371/journal.pone.0300983)
Supplement: S2 File — (DOCX) [file pone.0300983.s002.docx]

Health literacy scale for patients with gout

| Item | I don’t know at all | I know a little | I know something | I know a lot | I know fully |
| --- | --- | --- | --- | --- | --- |
| 1. I know gout is caused by the deposition of sodium urate crystals due to elevated blood uric acid levels. |  |  |  |  |  |
| 2. I know gout is divided into asymptomatic hyperuricemia, acute gouty arthritis, intermittent, chronic tophi, and chronic gouty arthritis. |  |  |  |  |  |
| 3. I know the target value of blood uric acid for patients with gout is less than 360 μmol/L (6 mg/dl). |  |  |  |  |  |
| 4. I know there is a genetic predisposition to gout. |  |  |  |  |  |
| 5. I know gout is an independent risk factor for cardiovascular and cerebrovascular diseases (e.g., hypertension, hyperlipidemia, stroke, coronary heart disease), diabetes mellitus, kidney stones, chronic kidney disease, etc. |  |  |  |  |  |
| 6. I know animal offal, crustacean seafood, thick meat soup, red meat (e.g., pork, beef, mutton), and alcoholic beverages are high in purines and should be restricted for patients with gout. |  |  |  |  |  |
| 7. I know people with gout should limit the consumption of fruits, fruit juices, beverages, snacks, and other foods rich in fructose or sucrose. |  |  |  |  |  |
| 8. I know purine-rich vegetables do not increase the risk of gout and should not be strictly limited for patients with gout. |  |  |  |  |  |
| 9. I know patients with gout should drink skimmed or low-fat milk or yogurt in moderation daily. |  |  |  |  |  |
| 10. I know patients with gout with normal kidney function should drink more than 2,000ml of water (e.g., light tea, unsweetened coffee) every day. |  |  |  |  |  |
| 11. I know patients with gout should limit the purine content of their diet to less than 200 mg per day. |  |  |  |  |  |
| 12. I know it is normal to have gouty attacks at the beginning of taking uric acid-lowering drugs (e.g., allopurinol, febuxostat), which means that the sodium urate crystals are being dissolved and cleared and that small doses of colchicine and other drugs can reduce gouty attacks if taken in accordance with the doctor's instructions. |  |  |  |  |  |
| 13. I know patients with gout usually need to take long-term or even lifelong medication for gout and that they still need to continue to take medicines according to the doctor's advice, even after their uric acid levels have dropped to normal. |  |  |  |  |  |
| 14. I know medications such as diuretics, vitamin B12, and combination antihypertensive tablets can reduce the body's ability to excrete uric acid. |  |  |  |  |  |
| 15. I know the names, effects, precautions, and side effects of commonly used medications for gout. |  |  |  |  |  |
| 16. I know low- to moderate-intensity aerobic exercise (e.g., walking, slow swimming, tai chi) for ≥30 minutes per day, ≥5 days per week, can reduce the risk of gouty attacks. |  |  |  |  |  |
| 17. I know high-intensity anaerobic exercise (e.g., fast running, basketball, hiking) can increase the risk of gouty attacks when blood uric acid levels are not within the standard range. |  |  |  |  |  |
| 18. I know sudden exposure to cold increases the risk of gouty attacks. |  |  |  |  |  |
| 19. I know braking and elevation of the affected limb can reduce pain during a gouty attack. |  |  |  |  |  |
| 20. I know I can apply cold compresses to relieve pain during a gouty attack. |  |  |  |  |  |
| Item | Not at all | rarely | sometimes | most often the times | always |
| 21. I can actively search for gout-related health information through books, the Internet, and other means. |  |  |  |  |  |
| 22. I am able to proactively ask healthcare professionals for gout-related health information. |  |  |  |  |  |
| 23. I am able to explain my condition clearly when communicating with healthcare professionals. |  |  |  |  |  |
| 24. I am able to understand gout-related health information they provide when communicating with healthcare professionals. |  |  |  |  |  |
| 25. I am able to seek help from others to explain gout-related health information that I do not understand. |  |  |  |  |  |
| 26. I am able to disseminate gout-related health information to fellow patients, family members, friends, and colleagues. |  |  |  |  |  |
| 27. I can implement the gout prevention and control information I have received. |  |  |  |  |  |
| 28. I will consider whether the sources of my gout-related health information are authoritative and reliable. |  |  |  |  |  |
| 29. I will consider whether the content of the gout-related health information I have received is correct. |  |  |  |  |  |
| 30. I am able to judge the authenticity of gout-related advertisements and promotions. |  |  |  |  |  |
| 31. I will think carefully about whether gout-related health information applies to my situation, even if it is reliable and of good quality. |  |  |  |  |  |
