# Supplementary material for: Health literacy in patients with gout: A latent profile analysis
Source: PLoS One. 2024 May 9;19(5):e0300983. doi: 10.1371/journal.pone.0300983 (PMC11081339; doi:10.1371/journal.pone.0300983)
Supplement: S3 File — (DOCX) [file pone.0300983.s003.docx]

痛风患者健康素养量表

| 条目 | 完全不知晓 | 少部分知晓 | 一般 | 大部分知晓 | 完全知晓 |
| --- | --- | --- | --- | --- | --- |
| 1.我知晓痛风是由血尿酸水平升高导致尿酸钠晶体沉积引发的。 |  |  |  |  |  |
| 2.我知晓痛风分为无症状高尿酸血症期、急性痛风性关节炎期、间歇期、慢性痛风石及慢性痛风性关节炎期。 |  |  |  |  |  |
| 3.我知晓痛风患者血尿酸的目标值为360μmol/L（6 mg/dl）以下。 |  |  |  |  |  |
| 4.我知晓痛风具有一定的遗传倾向。 |  |  |  |  |  |
| 5.我知晓痛风是心脑血管疾病（如高血压、高脂血症、脑卒中、冠心病）、糖尿病、肾结石、慢性肾病等疾病的独立危险因素。 |  |  |  |  |  |
| 6.我知晓动物内脏、甲壳类海鲜，浓肉汤、红肉（如猪肉、牛肉、羊肉）、酒类饮料嘌呤含量高，痛风患者应限制食用。 |  |  |  |  |  |
| 7.我知晓痛风患者应限制食用富含果糖或蔗糖的水果、果汁、饮料、点心等食物。 |  |  |  |  |  |
| 8.我知晓富含嘌呤的蔬菜不会增加痛风的发病风险，痛风患者不必严格限制食用。 |  |  |  |  |  |
| 9.我知晓痛风患者每日应适量饮用脱脂或低脂的牛奶或酸奶。 |  |  |  |  |  |
| 10.我知晓肾功能正常的痛风患者应每日饮水（包含淡茶和不加糖的咖啡）2000ml以上。 |  |  |  |  |  |
| 11.我知晓痛风患者每日饮食嘌呤含量应控制在200 mg以下。 |  |  |  |  |  |
| 12.我知晓服用降尿酸药物(如别嘌呤醇、非布司他等)初期会引起痛风发作是正常的，代表尿酸钠晶体正在溶解破碎与清除，遵医嘱预防性服用小剂量秋水仙碱等药物可减少痛风发作。 |  |  |  |  |  |
| 13.我知晓痛风患者一般需要长期甚至终生服用治疗痛风的药物，尿酸降至正常后仍需根据医嘱继续服药。 |  |  |  |  |  |
| 14.我知晓一些药物如利尿剂、维生素b12、复方降压片等会降低机体排泄尿酸的能力。 |  |  |  |  |  |
| 15.我知晓痛风常用药物的名称、作用、注意事项和副反应等。 |  |  |  |  |  |
| 16.我知晓每周≥5 天、每天单次≥30 分钟低中强度的有氧运动（如散步、慢速游泳、打太极拳等）可降低痛风发作的风险。 |  |  |  |  |  |
| 17.我知晓在血尿酸未达标时，高强度的无氧运动（如快跑、打篮球、登山等）可增加痛风发作的风险。 |  |  |  |  |  |
| 18.我知晓突然受凉可增加痛风发作的风险。 |  |  |  |  |  |
| 19.我知晓痛风发作时可通过患肢制动、患肢（下肢）抬高来减轻疼痛。 |  |  |  |  |  |
| 20.我知晓痛风发作时可通过局部冷敷来减轻疼痛。 |  |  |  |  |  |
| 条目 | 没有 | 很少 | 有时 | 经常 | 总是 |
| 21.我能够通过书籍、网络等方式主动搜寻痛风相关健康信息。 |  |  |  |  |  |
| 22.我能够主动向医护人员询问痛风相关健康信息。 |  |  |  |  |  |
| 23.与医护人员交流时，我能够向医护人员介绍清楚自己的病情。 |  |  |  |  |  |
| 24.与医护人员交流时，我能够理解医护人员所述痛风相关健康信息。 |  |  |  |  |  |
| 25.遇到不理解的痛风相关健康信息，我能够寻求他人帮忙解释。 |  |  |  |  |  |
| 26.我能够向病友、家人、朋友、同事传播痛风相关健康信息。 |  |  |  |  |  |
| 27.我能够实施我获得的有关痛风发作预防和控制的健康信息。 |  |  |  |  |  |
| 28.我会思考我所获得的痛风相关健康信息的来源是否权威、可靠。 |  |  |  |  |  |
| 29.我会思考我所获得的痛风相关健康信息的内容是否正确。 |  |  |  |  |  |
| 30.我能够判断出痛风相关广告宣传的真伪。 |  |  |  |  |  |
| 31.即使是可靠的、高质量的痛风相关健康信息，我也会认真思考它是否适用于我的个人情况。 |  |  |  |  |  |
